# Safety and efficacy of triple combination therapy in hypertension and dyslipidemia: a systematic review and meta-analysis of randomized controlled trials

**DOI:** 10.1186/s43044-026-00720-z

**Published:** 2026-02-16

**Authors:** Ahmed Yasser Shaban, Ahmed Hassan, Sherif Eltawansy, Malak A. Hassan, Mariam Tarek Desouki, Nourhan Ahmed, Salem Badr, Akram Y. Elgendy, Mahmoud Nassar, Diaa Hakim

**Affiliations:** 1 Kafr El-Sheikh University, Kafr El-Sheikh, Egypt; 2 Suez Medical Complex, Ministry of Health and Population, Suez, Egypt; 3 Jersey Shore University Medical Center, Neptune City, USA; 4 Alexandria University, Alexandria, Egypt; 5 Minneapolis Heart Institute Foundation, Minneapolis, USA; 6 Aurora St. Luke’s Medical Center, Milwaukee, USA; 7 University Hospitals Cleveland Medical Center, Harrington Heart and Vascular Institute, Cleveland, USA; 8 Division of Endocrinology and Diabetes, Larner College of Medicine, University of Vermont, Burlington, USA; 9 Brigham and Women’s Hospital/Harvard Medical School, Boston, USA

**Keywords:** Hypertension, Dyslipidemia, Triple therapy, Dual therapy, Meta-analysis

## Abstract

**Background:**

Hypertension (HTN) and dyslipidemia are major risk factors for cardiovascular diseases. Recently, researchers have investigated the potential benefits of combining multiple medications in one bill to improve their metabolic and cardiovascular efficacy.

**Objectives:**

We investigated a treatment approach that combines two antihypertensive medications with one statin. We aim to assess the safety and effectiveness of a triple therapy regimen consisting of angiotensin receptor blockers (ARBs) combined with amlodipine/rosuvastatin. We compared this triple therapy to dual therapy involving either ARBs/amlodipine or ARBs/rosuvastatin in patients with HTN and dyslipidemia.

**Methods:**

We conducted systematic search in the following databases: Medline, Web of Science, Scopus, and Cochrane Library until August 2024. The main outcomes assessed were the variations in mean systolic blood pressure (mSBP), mean diastolic blood pressure (mDBP), and the percentage changes in LDL cholesterol (LDL-C) and HDL cholesterol (HDL-C) following an eight-week treatment period.

**Results:**

Our analysis included seven randomized controlled trials (RCTs) which enrolled 1074 patients. Triple therapy revealed a significant reduction in mSBP (mean difference (MD): -4.06, 95% C.I. [-7.97: -0.15], p-value = 0.04), mDBP (MD: -5.45, 95% C.I. [-7.96: -2.93], p-value = < 0.0001), and LDL-C (MD: -50.10, 95% C.I. [-55.55: -44.64], p-value = < 0.001) compared to ARBs/amlodipine. Triple therapy significantly decreased mSBP (MD: −12.28, 95% C.I. [− 16.68: −7.88], p-value = < 0.001) and mDBP levels (MD: −6.48, 95% C.I. [-10.95: -2.01], p-value = 0.005) compared with ARBs plus rosuvastatin. There was no significant difference in secondary outcomes, including total adverse events, cerebrovascular adverse events, and adverse drug reactions.

**Conclusion:**

Triple therapy has greater effectiveness in decreasing blood pressure in hypertensive patients with dyslipidemia compared to treatments involving ARBs combined with amlodipine or ARBs with rosuvastatin. Additionally, Triple therapy significantly improved lipid profiles compared to the ARB/amlodipine group. Our study lays the groundwork for developing a single-pill, triple-combination therapy. Further RCTs are necessary to confirm our findings.

**Supplementary Information:**

The online version contains supplementary material available at 10.1186/s43044-026-00720-z.

## Introduction

Cardiovascular disease (CVD) is considered one of the most common causes of morbidity and mortality worldwide [1]. A significant percentage of patients diagnosed with hypertension (HTN) concurrently exhibit dyslipidemia [2, 3]. Piepoli et al. have highlighted a direct correlation between blood cholesterol and CVD, emphasizing the importance of reducing cholesterol to effectively lower CVD risk [4]. As a result, recent HTN management has focused not only on lowering blood pressure but also on reducing overall CVD risk and this can be achieved by lifestyle modification and optimizing medication regimens to improve patient compliance and improve clinical outcomes [3, 5]. In 2017, the American College of Cardiology and American Heart Association published guidelines on hypertension treatment in adults which recommend combining two antihypertensive drugs from different classes for managing second-stage hypertension, defined as a blood pressure (BP) > = 140/90 mmHg [6]. The guidelines recommend ARBs combined with calcium channel blockers (CCBs) as a first-line treatment among the various available drug combinations.

Rosuvastatin is an effective and safe option for treating dyslipidemia [7]. However, many patients continue to experience uncontrolled dyslipidemia, which further increases their cardiovascular risk. While previous meta-analyses have focused on comparing the efficacy of various pharmacological interventions for HTN, they have given limited attention to the coexistence of dyslipidemia [8, 9].

The optimal treatment strategy should tackle all these factors simultaneously, once-daily dosing and fixed-dose combination therapy can maintain good control of both BP and cholesterol levels.

Hence, we perform this systematic review and meta-analysis to assess the safety and efficacy of triple combination therapy with ARBs/amlodipine plus rosuvastatin compared with ARBs/amlodipine or ARBs/rosuvastatin in patients with HTN and dyslipidemia.

## Method

### Search strategy and data collection

We systematically searched PubMed/Medline, Web of Science, Scopus, and the Cochrane Library for relevant studies up to August 2024 using the following search terms: (“Telmisartan” OR “Pritor” OR “BIBR” OR “Micardis” OR “Kinzalmono” OR “Semintra” OR “Tolura”) OR (“Amlodipine” OR “Amlodis” OR “Norvasc” OR “Istin” OR “Amlor”) OR (“Rosuvastati” OR “Crestor” OR “Ezallor”) AND (“Dyslipidemia” OR “Dyslipoproteinemia*” OR “Hyperlipidemia*” OR “Hypercholesterolemia*” OR “Hyperlipoproteinemia*” OR “Hypertriglyceridemia*” OR “Atherosclerosis”) AND (“Hypertension” OR “High Blood Pressure”) (Supplementary Table 1). To ensure comprehensiveness, manual searches were also performed by the first four authors using alternative terms, including “losartan,” “valsartan,” and “olmesartan,” while focusing the strategy to minimize irrelevant studies.

We used Endnote software (Clarivate Analytics, PA, USA) for duplicate removal. The screening of the retrieved studies was conducted in 2 stages: First, AH, MTD, HMA, and NA independently screened the titles and abstracts to evaluate their relevance. Next, the full text of the selected abstracts was screened to determine if they fulfilled the inclusion and exclusion criteria. We used the Rayyan website [10] to facilitate the screening process.

Studies including the following inclusion criteria were included: (1) Studies that enrolled patients with both HTN and dyslipidemia or hypercholesterolemia; (2) Studies comparing triple therapy with ARBs/amlodipine plus rosuvastatin versus dual therapies with ARBs/amlodipine or ARBs with rosuvastatin; and (3) Studies that reported our search outcomes and were published in international peer-reviewed journals. We excluded non-English studies, abstracts without available data, Literature reviews, preclinical studies, and pharmacokinetics/pharmacodynamics studies.

A fourth co-author independently extracted the data to MS excel sheet, with any conflicts regarding study inclusion resolved by a fifth co-author. We organized the extracted data into three domains: (1) summary of study characteristics, (2) baseline characteristics of the study population, and (3) study outcomes.

### Outcomes

Primary outcome measures were the differences in mean systolic and diastolic blood pressure (mSBP, mDBP) and the relative changes in LDL and HDL cholesterol (LDL-C, HDL-C) after eight weeks of treatment. Secondary outcomes included overall adverse events, cerebrovascular adverse events, and adverse drug reactions.

### Risk of bias assessment

The revised Cochrane risk-of-bias tool (RoB2) was used to evaluate the risk of bias in the included randomized controlled trials (RCTs) [11]. It included the process of randomization, concealment, deviations from intended interventions, use of appropriate analysis to assess the intervention, outcome measurement, selection of reported overall risk of bias and results. The methodological quality of the studies was categorized as either low risk, some concerns, or high risk of bias. Two independent authors assessed the risk of bias, and disagreements were resolved through discussion with a third author.

### Statistical analysis

We used RevMan v5.3 for statistical analysis [12]. Continuous outcomes were presented as mean difference (MD) with corresponding 95% confidence intervals (C.I.), while categorical outcomes were presented as risk ratios (R.R) with corresponding 95% C.I., which describe the ratio of the risk of an outcome event between the two groups. A fixed-effect model was applied for homogeneous outcomes; however, a random-effect model was utilized when significant heterogeneity was detected. I² and Chi-square tests were used to assess heterogeneity, where Chi-square determines the presence and degree of heterogeneity. I² values were interpreted according to the Cochrane Handbook (Chap. 9) [13] as follows: 0–40% (not significant), 30–60% (moderate), 50–90% (substantial), and 75–100% (considerable). A significance level (α) below 0.1 in the Chi-square test was considered indicative of significant heterogeneity, as per the Cochrane Handbook (Part 2, Chap. 9) [13]. Heterogeneity in some outcomes was resolved using a random-effect model and conducting a sensitivity analysis. We excluded one study at a time and generated forest plots for the remaining studies to achieve the lowest possible heterogeneity. For dealing with missing data, when the standard deviation (SD) of change in outcomes was not provided, we calculated it from the standard error (SE) or 95% CI, as per Altman’s methods [14].

### Publication bias

A test for publication bias was not conducted, as tests for funnel plot asymmetry are recommended only for the outcome reported in at least ten studies, according to Cochrane guidelines [13].

## Results

**Fig. 1:**
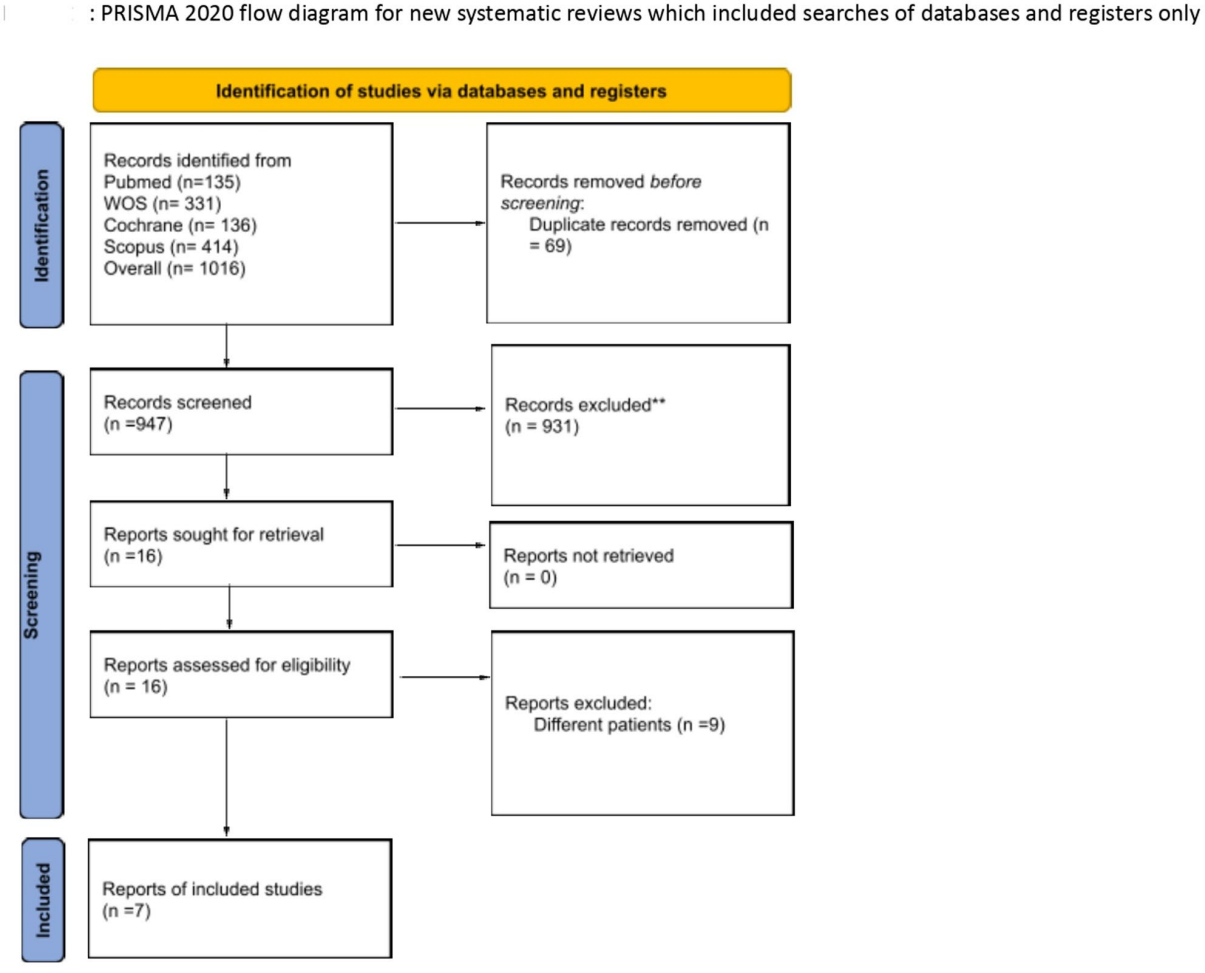
PRISMA flow diagram of the included studies

We screened 1,016 articles through our search, with 947 remaining after removing duplicates. Following the title and abstract screening, we selected 16 articles for full-text screening. Out of these, seven studies were finally included [5, 15–20]. The PRISMA flow diagram illustrates the selection process in Fig. 1, and the study is registered on PROSPERO (CRD42024570094). Six studies were assessed as having a low risk of bias, while one study showed some concerns, particularly related to selection bias (Supplementary Fig. 1). The characteristics of the included studies are detailed in Supplementary Table 2.

### Baseline characteristics

We included 1,074 patients, 67.6% of them were male. The group receiving triple therapy had a mean age of 64.50 ± 9.58 years, with a mean SBP of 152.15 ± 12.72 mmHg and a mean LDL-C level of 154.97 ± 1.39 mg/dL. In comparison, the cohort treated with ARBs/amlodipine had a mean age of 63.75 ± 9.91 years, a mean SBP of 150.70 ± 11.87 mmHg, and a mean LDL-C of 154.91 ± 31.20 mg/dL. Meanwhile, the group receiving ARBs with rosuvastatin had a mean age of 63.25 ± 9.30 years, a mean SBP of 151.62 ± 12.52 mmHg, and a mean LDL-C of 156.85 ± 32.66 mg/dL. Four studies administered triple combination therapy in the form of three tablets, while two studies used two tablets—one combining ARBs/amlodipine and the other containing rosuvastatin. In contrast, one study (Kim et al., 2023) used a single tablet for triple therapy.

Additional baseline characteristics are described in Table 1.

**Table 1:** Baseline characteristics of included studies (T1)

Study ID	Arms	*N*	Patient demographics				Risk factor		Laboratory parameters			
			Age (y) mean ± SD	Male *N* (%)	BMI (kg/m2) mean ± SD	DM, *N* (%)	Smoking Status *N* (%)		Total Cholesterol (mg/dL) Mean ± SD	LDL-C (mg/dL) Mean ± SD	HDL-C (mg/dL) Mean ± SD	SBP (mmHg) Mean ± SD
							Current	Former				
Kim 2023 [15]	Olmesartan/ Amlodipine/ Rosuvastatin 20/5/5 mg	32	59.2 ± 9.8	20 (62.5)	25.7 ± 2.3	-	3 (12.5)	-	-	-	-	-
	Amlodipine / Olmesartan 20/5 mg	34	60.9 ± 11.8	23 (67.6)	25.6 ± 2.6	-	10 (32.3)	-	-	-	-	-
Jeon 2022 [16]	fmasartan/ Amlodipine + Rosuvastatin 60/10/20 mg	43	59.6 ± 7.4	32 (74.4)	-	-	30 (57.7)	15 (34.9)	221.8 ± 28.4	157.21 ± 27.45	48.4 ± 13.7	154 ± 10.3
	fmasartan /Amlodipine 60/10 mg	45	60.8 ± 6.3	36 (8)	-	-	12 (26.7)	16 (35.6)	213.5 ± 35.6	151.22 ± 32.86	45.9 ± 12.1	152.9 ± 8.7
	fmasartan + Rosuvastatin 60/20 mg	43	60.7 ± 6.8	30 (69.8)	-	-	12 (27.9)	9 (20.9)	223.8 ± 40.1	157.95 ± 36.43	48.8 ± 10.6	152.5 ± 8.6
Jo 2022 [17]	Olmesartan/Amlodipine (SPC) + Rosuvastatin 40/10/20 mg	105	65.18 ± 9.34	59 (56.2)	26.75 ± 3.3	-	21 (20)	27 (25.7)	216.98 ± 34.8	154.52 ± 30.84	49.23 ± 11.9	153.58 ± 10.9
	Olmesartan /Amlodipine 40/10 mg	52	64.06 ± 8.9	31 (59.6)	26.67 ± 3.3	-	10 (19.2)	12 (23.1)	223.48 ± 37.2	-	48.65 ± 10.7	151.30 ± 8.9
	Olmesartan + Rosuvastatin 40/20 mg	102	63.49 ± 9.8	58 (56.9)	26.60 ± 3	-	26 (25.5)	20 (19.6)	220.63 ± 35.2	160.42 ± 32.05	46.87 ± 11.5	153.71 ± 11.1
Jin 2020 [18]	Telmisartan/ Amlodipine+ Rosuvastatin 80/5/20 mg	66	68.33 ± 8.3	45 (68.2)	26.26 ± 3.4	28 (42.42)	10 (15.15)	-	225.55 ± 34.3	160.12 ± 32.34	46.38 ± 10.9	155.40 ± 12.1
	Telmisartan/Amlodipine 80/5 mg	66	66.01 ± 10.43	48 (72.7)	26.67 ± 2.8	29 (43.94)	15 (22.73)	-	218.17 ± 36.9	153.41 ± 31.30	47.80 ± 12	155 ± 12.1
	Telmisartan+ Rosuvastatin 80/20 mg	65	64.9 ± 9.11	46 (70.8)	26.60 ± 3.2	32 (49.23)	11 (16.92)	-	221.20 ± 38.7	153.88 ± 36.73	47.89 ± 12.9	154.42 ± 12.9
Hong 2019 [19]	Telmisartan/ Amlodipine+ Rosuvastatin 80/10/20 mg	47	67.96 ± 9.44	33 (70.21)	26.52 ± 3.12	15 (31.91)	-	-	216.38 ± 31.68	150.51 ± 31.78	46.17 ± 12.36	149.49 ± 12.09
	Telmisartan/Amlodipine 80 mg/10 mg	49	66.63 ± 10.22	31 (63.27)	26.55 ± 2.92	16 (32.65)	-	-	219.84 ± 37.88	153.12 ± 35.18	49.76 ± 13.60	144.29 ± 11.09
	Telmisartan + Rosuvastatin 80/ 20 mg	48	65.88 ± 9.13	34 (70.83)	27.67 ± 3.41	13 (27.08)	-	-	225.69 ± 32.84	158.92 ± 30.62	47.44 ± 11.98	147.08 ± 13.69
Kim 2019 [20]	Telmisartan/Amlodipine+ Rosuvastatin 80/10/20 mg	41	67.4 ± 10.0	29 (72.5)	-	15 (37.5)	-	-	223.5 ± 36.1	155 ± 29.2	50.2 ± 14.5	156.8 ± 13.7
	Telmisartan/Amlodipine 80/10 mg	44	66.4 ± 11.0	27 (62.8)	-	16 (37.2)	-	-	221.6 ± 28.9	155.7 ± 23.1	46.3 ± 10.7	154.8 ± 10.6
	Telmisartan+ Rosuvastatin 80/20 mg	49	63.4 ± 9.7	37 (75.5)	-	15 (30.6)	-	-	228.5 ± 36.5	160 ± 32	46.9 ± 10.3	154.8 ± 10.6
Lee 2017 [21]	Amlodipine/ Losartan Potassium/ Rosuvastatin 5/100/ 20 mg	54	60.33 ± 8.48	37 (68.52)	26.36 ± 3.08	-	-	-	219.91 ± 38.20	151.63 ± 35.59	46.72 ± 11.43	142.74 ± 13.64
	Amlodipine/ Losartan Potassium 5/100 mg.	43	59.28 ± 7.44	35 (81.40)	26.47 ± 2.85	-	-	-	215.49 ± 29.75	162.33 ± 31.86	45.33 ± 11.29	144.15 ± 14.04
	Losartan Potassium/ Rosuvastatin 100/20 mg	46	59.83 ± 9.09	35 (76.09)	27.50 ± 3.88	-	-	-	235.11 ± 38.62	146.58 ± 25.36	46.26 ± 10.53	143.54 ± 14.39

Data are presented as mean ± SD or n (%).Body mass index, (BMI); Diabetes mellitus, (DM); High-density lipoprotein-cholesterol, (HDL-C); Low-density lipoprotein-cholesterol (LDL-C); Systolic blood pressure, (SBP); Single pill combination, (SPC).

### Clinical outcomes

#### Triple therapy vs. ARBs/Amlodipine

##### Change in mSBP

**Fig. 2:**
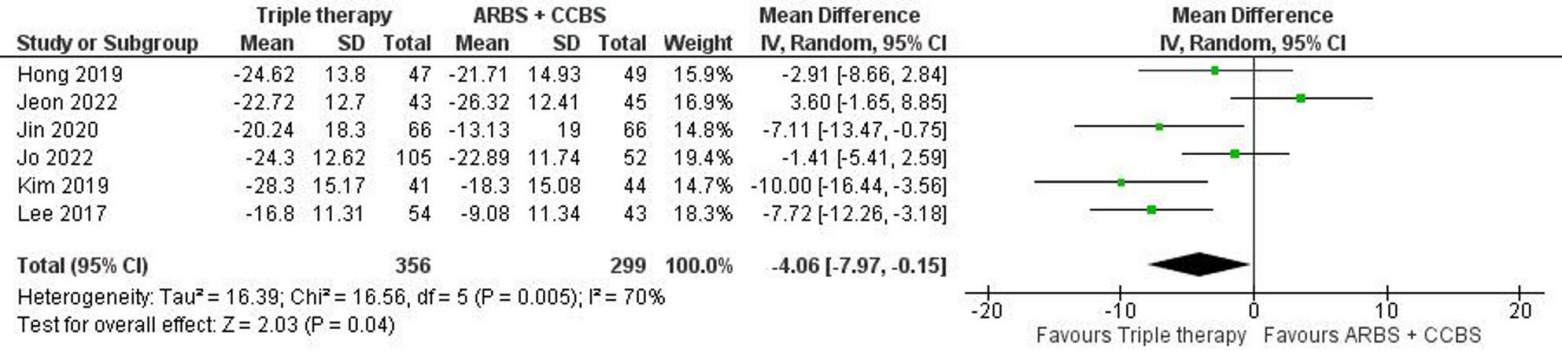
Forest plot of change in mean systolic blood pressure (mSBP) (Triple therapy vs. ARBs/amlodipine)

Our analysis included six studies with 356 patients in the triple therapy group and 299 in the ARBs/amlodipine group. The results showed a significant difference in favor of the triple therapy group (MD: -4.06, 95% C.I. [-7.97: -0.15], p-value = 0.04) (Fig. 2). However, we observed considerable heterogeneity (*P* = 0.005, I² = 70%), which was resolved by excluding the study by Jeon et al., 2022 (Figure S2).

##### Change in mDBP

**Fig. 3:**
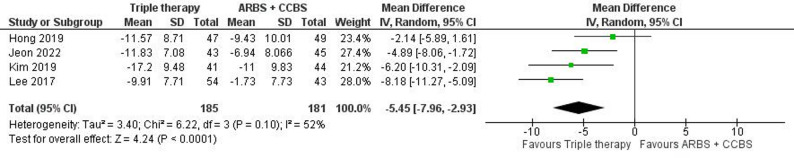
Forest plot of change in mean diastolic blood pressure (mDBP) (Triple therapy vs. ARBs/amlodipine)

The triple therapy group (181 participants) showed a significant reduction in mDBP (MD: -5.45, 95% C.I. [ -7.96: -2.93], p-value < 0.0001) compared with the ARBs/amlodipine group (Fig. 3). Moderate heterogeneity was found (*P* = 0.10, I² = 52%), which was addressed by removing the Hong, 2019 study (Figure S3).

##### Change in LDL-C

**Fig. 4:**
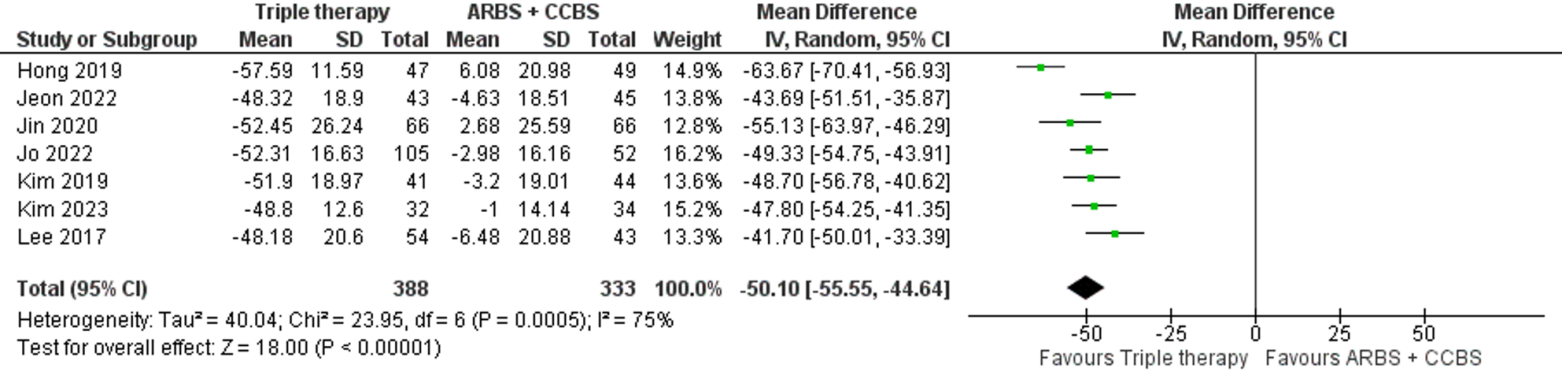
Forest plot of mean percentage change in LDL-C (Triple therapy vs. ARBs/ amlodipine)

This outcome was reported in seven studies with 388 participants in the triple therapy group and 333 in the ARBs/amlodipine group. A significant reduction in LDL-C was reported in the triple therapy group (MD: -50.10, 95% C.I. [-55.55: -44.64], p-value < 0.00001) (Fig. 4). Heterogeneity was significant (I² = 75%), but resolved by excluding Hong, 2019 (Figure S4).

##### Change in HDL-C and triglycerides (TGs)

**Fig. 5:**
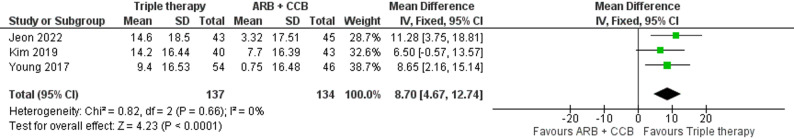
Forest plot of mean percentage change in HDL-C (Triple therapy vs. ARBs/ amlodipine)

Three studies with 137 participants in the triple therapy group and 134 in the ARBs/amlodipine group found a significant increase in HDL-C but no significant difference in triglyceride levels (MD: 8.7, 95% C.I. [4.67: 12.74], p-value < 0.0001), (MD: -6.89, 95% C.I. [-15.75 to 1.96], p-value = 0.13), respectively (Fig. 5, S5).

##### Safety outcomes

The two groups showed no statistically significant differences regarding total adverse events and adverse drug reactions (R.R: 1. 09, 95% C.I. [0.73: 1.63], p-value = 0.66) and (R.R: 1.03, 95% C.I. [0.59: 1.78], p-value = 0.93), respectively (Figure S6, S7). For CNS adverse events, data from four studies also showed no significant differences (R.R: 1.18, 95% C.I. [0.34, 4.09], p-value = 0.79), (Figure S8).

All clinical outcomes were assessed over a duration of 8 weeks with no heterogeneity in the change in HDL-C, change in TG, or any of the safety outcomes between the two groups.

### Triple therapy vs. ARBs with Rosuvastatin

#### Change in mSBP

**Fig. 6:**
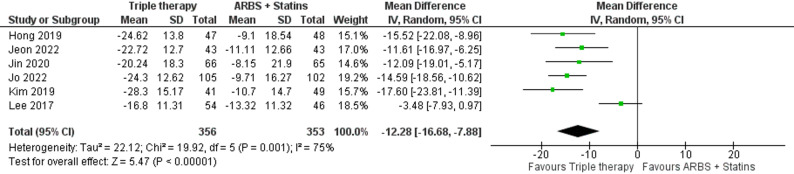
Forest plot of change in mean systolic blood pressure (mSBP) (Triple therapy vs. ARBs with rosuvastatin)

In a comparison of six studies with 356 patients in the triple therapy group and 353 in the ARBs with rosuvastatin group, the triple therapy showed a significant reduction in mSBP (MD: -12.28, 95% C.I. [-16.68: -7.88], p-value < 0.00001) (Fig. 6, though there was significant heterogeneity resolved by excluding the study by Lee, 2017 (Figure S9).

#### Change in mDBP

**Fig. 7:**
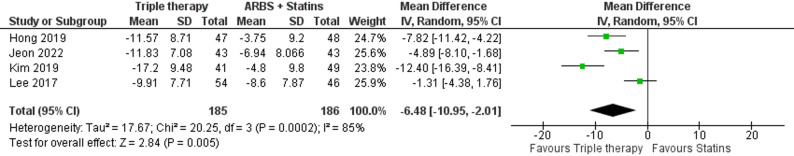
Forest plot of change in mean diastolic blood pressure (mDBP) (Triple therapy vs. ARBs with rosuvastatin)

There was a significant reduction in mDBP when comparing 185 participants in the triple therapy group with 186 in the ARBs with rosuvastatin group that favores the triple therapy (MD: -6.48, 95% C.I. [-10.95: -2.01], p-value = 0.005) (Fig. 7). However, removing the Kim, 2019 study reduced the heterogeneity (*P* = 0.0002, I² = 85%), though it did not resolve it completely (Figure S10).

#### Change in LDL-C

**Fig. 8:**
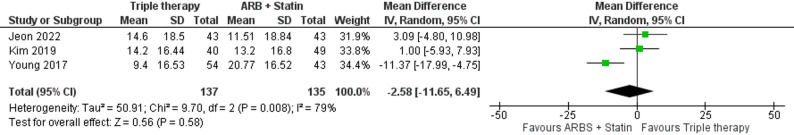
Forest plot of mean percentage change in LDL-C (Triple therapy vs. ARBs with rosuvastatin)

We did not notice any significant difference in LDL-C reduction between the two groups in the included Five studies with 251 participants in the triple therapy group and 247 in the ARBs with rosuvastatin (MD: -2.07, 95% C.I. [-5.17: 1.04], p-value = 0.19) (Fig. 8).

#### Change in HDL-C and triglycerides (TGs)

**Fig. 9:**
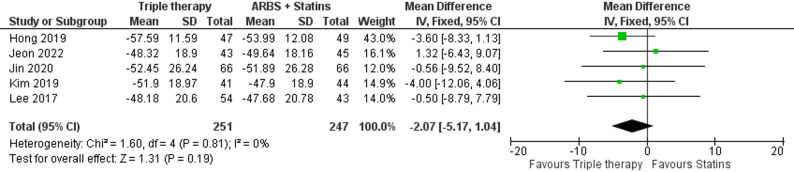
Forest plot of mean percentage change in HDL-C (Triple therapy vs. ARBs with rosuvastatin)

The analysis of three studies with 137 patients in the triple therapy group and 135 in the ARBs with rosuvastatin group found no significant difference in HDL-C levels and TGs levels (MD: -2.58, 95% C.I. [-11.65: 6.49], p-value = 0.58), (MD: 1.91, 95% C.I. [-6.64: 10.47], p-value = 0.66), respectively. (Fig. 9, S12). Heterogeneity was notable in HDL-C change, which was resolved by excluding the study by Young, 2017 (Figure S11).

#### Safety outcomes

In our analysis, five studies reported total adverse events, showing no difference between the groups (R.R: 1. 06, 95% C.I. [0.71: 1.58], p-value = 0.79) (Figure S13). Similarly, there were no differences in adverse drug reactions (R.R: 1.41, 95% C.I. [0.77: 2.58], p-value = 0.27) (Figure S14) or CNS adverse events (R.R: 0.82, 95% C.I. [0.28: 2.40], p-value = 0.72) (Figure S15).

All clinical outcomes were evaluated over 8 weeks, with no heterogeneity observed in the changes in LDL-C, triglycerides, or any safety outcomes between the two groups.

## Discussion

Our meta-analysis included 1,074 patients from seven RCTs and evaluated the effects of triple therapy (ARBs/amlodipine plus rosuvastatin) compared with double therapy (ARBs/amlodipine or ARBs plus rosuvastatin) on blood pressure, lipid profiles, and safety outcomes. Triple therapy significantly reduced mSBP and mDBP compared to both double therapy groups and was associated with notable improvements in LDL-C and HDL-C levels, particularly compared to the ARB/amlodipine group. Importantly, our study also assessed the safety of triple therapy versus double therapy, revealing promising results suggesting that both approaches are similarly safe. No significant differences were observed in TG levels across the comparisons, and triple therapy showed comparable lipid effects to ARBs plus rosuvastatin.

The prevalence of HTN is rising alongside dyslipidemia, with up to 80% of patients experiencing both conditions simultaneously [21, 22]. Antihypertensive therapy can effectively reduce cardiovascular events—such as myocardial infarction, and heart failure—by 20% to 50% [23]. Clinical guidelines strongly recommend statins for lowering LDL-C in CVD prevention [24]. However, significant treatment gaps persist in clinical practice. Only half of hypertensive patients receive treatment, with just 34% achieving adequate blood pressure control [25]. Similarly, statins and lipid-modifying agents remain underutilized in high-risk populations, contributing to preventable cardiovascular events [26, 27]. Compliance with statin therapy is also suboptimal, with 60% of patients ceasing treatment within the initial year and only 25% reaching their LDL-C targets [28, 29]. Non-compliance with prescribed medications significantly contributes to these outcomes, leading to treatment failures and widening the disparity between clinical recommendations and actual results [30]. The complexity of managing multiple medications often overwhelms patients, especially those dealing with several health conditions or older individuals, resulting in poor adherence rates.

Previous studies have shown that statin therapy can lower blood pressure which might be attributed to its pleotropic effect or other mechanisms [31–34]. For instance, a large meta-analysis involving around 50,000 patients from 46 clinical trials [32] found that statins, regardless of type or dosage, effectively reduce blood pressure, especially in younger individuals. These findings support the use of statins in combination with blood pressure medications, as seen in our study, where a statin was administered alongside two blood pressure medications. It’s worth noting that our study also assessed the safety of triple therapy versus double therapy, revealing promising results that suggest both approaches are similarly safe. However, a major drawback of triple therapy is its higher cost compared to monotherapy, along with potential challenges from drug interactions, which may complicate the interpretation of reported side effects.

Considering the close association between HTN and dyslipidemia—both are major risk factors for CVD—earlier investigations have explored the efficacy of integrating antihypertensive agents with lipid-lowering medications, such as statins [35, 36]. A prior meta-analysis [37] encompassing six RCTs demonstrated favorable outcomes for a triple therapeutic regimen comprising ACE inhibitors, diuretics, and calcium channel blockers, resulting in enhanced blood pressure regulation without compromising safety. Furthermore, a recent meta-analysis [38] underscored the critical role of triple antihypertensive treatment in individuals with high-risk hypertension. Consequently, we advocate for a novel therapeutic strategy that combines two antihypertensive drugs with a single statin. This approach is potentially beneficial for the proper treatment of both elevated blood pressure and dyslipidemia. While previous studies have investigated triple therapy, our analysis uniquely focuses on evaluating the efficacy of the combination of two antihypertensive medications alongside one statin.

Although the included studies vary in the number of pills administered, our hypothesis suggests that reducing tablet quantity could significantly boost patient compliance with prescribed medications, potentially leading to improved cardiovascular metrics. As such, we emphasize additional RCTs that evaluate the efficacy of triple therapy, consolidated into one pill, compared to dual therapy in patients diagnosed with hypertension and dyslipidemia. This approach aligns with the current trend of combining multiple classes of antihypertensive drugs into a single tablet [39, 40].

### Strength and limitations

Our study stands as the first systematic review and meta-analysis assessing the efficacy and safety of triple therapy combining ARBs, amlodipine, and rosuvastatin in patients with hypertension and dyslipidemia, providing robust evidence on both blood pressure reduction and lipid profile improvement. All included studies were multicenter RCTs with no patient overlap; most were double-blind, except Kim et al. (2023), which was open label.

However, several limitations should be noted. All studies were conducted in Korea, potentially limiting generalizability to other populations. There was variability in drug dosages and formulations across trials, and sample sizes were relatively small. Additionally, patient adherence and compliance were not reported, which may affect real-world applicability.

## Conclusion

Our meta-analysis highlights that triple combination therapy with ARBs/amlodipine and rosuvastatin significantly lowers blood pressure, improves lipid profiles, and is similarly safe compared to double therapy in patients with hypertension and dyslipidemia. Future studies should focus on developing a single-pill triple-combination therapy and evaluating its impact on adherence and clinical outcomes.

## Supplementary Information

Below is the link to the electronic supplementary material.

Supplementary Material 1

## Data Availability

No datasets were generated or analysed during the current study.

## Abbreviations

ARBs: Angiotensin receptor blockers
ACE: Angiotensin converting enzyme
BP: Blood pressure
CVD: Cardiovascular disease
CI: Confidence intervals
HDL-C: High-density lipoprotein-cholesterol
HTN: Hypertension
LDL-C: Low-density lipoprotein-cholesterol
mSBP: Mean systolic blood pressure
mDBP: Mean diastolic blood pressure
MD: Mean difference
RCTs: Randomized controlled trials
RoB2: Revised Cochrane risk-of-bias tool
SD: Standard deviation
SE: Standard error
TGs: Triglycerides

## References

[1] Byass P (2016) Cause-specific mortality findings from the global burden of disease project and the INDEPTH network. Lancet Global Health 4(11):e785–e786. 10.1016/S2214-109X(16)30203-0 27765285 10.1016/S2214-109X(16)30203-0

[2] Noh J, Kim HC, Shin A et al (2016) Prevalence of comorbidity among people with hypertension: the Korea National health and nutrition examination survey 2007–2013. Korean Circ J 46(5):672. 10.4070/kcj.2016.46.5.672 27721859 10.4070/kcj.2016.46.5.672 PMC5054180

[3] Thakur S, Raina S, Thakur S, Negi P, Verma B (2013) Prevalence of metabolic syndrome among newly diagnosed hypertensive patients in the hills of Himachal Pradesh, India. Indian J Endocr Metab 17(4):723. 10.4103/2230-8210.113768 10.4103/2230-8210.113768 PMC3743377 23961493

[4] Piepoli MF, Hoes AW, Agewall S et al (2016) European Guidelines on cardiovascular disease prevention in clinical practice: The Sixth Joint Task Force of the European Society of Cardiology and Other Societies on Cardiovascular Disease Prevention in Clinical Practice (constituted by representatives of 10 societies and by invited experts)Developed with the special contribution of the European Association for Cardiovascular Prevention & Rehabilitation (EACPR). *Eur Heart J*. 2016;37(29):2315–2381. 10.1093/eurheartj/ehw106 10.1093/eurheartj/ehw106 PMC4986030 27222591

[5] Lee HY, Kim SY, Choi KJ et al (2017) A Randomized, Multicenter, Double-blind, Placebo-controlled study to evaluate the efficacy and the tolerability of a triple combination of Amlodipine/Losartan/Rosuvastatin in patients with comorbid essential hypertension and hyperlipidemia. Clin Ther 39(12):2366–2379. 10.1016/j.clinthera.2017.10.013 29150250 10.1016/j.clinthera.2017.10.013

[6] Whelton PK, Carey RM, Aronow WS et al (2018) 2017 ACC/AHA/AAPA/ABC/ACPM/AGS/APhA/ASH/ASPC/NMA/PCNA guideline for the Prevention, Detection, Evaluation, and management of high blood pressure in adults: executive summary: A report of the American college of Cardiology/American heart association task force on clinical practice guidelines. Hypertension 71(6):1269–1324. 10.1161/HYP.0000000000000066 29133354 10.1161/HYP.0000000000000066

[7] Kones R. Rosuvastatin, inflammation, C-reactive protein, JUPITER, and primary prevention of cardiovascular disease – a perspective. *DDDT*. Published online December 2010:383. doi:10.2147/DDDT.S10812 10.2147/DDDT.S10812 PMC3023269 21267417

[8] Wald DS, Law M, Morris JK, Bestwick JP, Wald NJ (2009) Combination therapy versus monotherapy in reducing blood pressure: Meta-analysis on 11,000 participants from 42 trials. Am J Med 122(3):290–300. 10.1016/j.amjmed.2008.09.038 19272490 10.1016/j.amjmed.2008.09.038

[9] Weisser B, Predel HG, Gillessen A et al (2020) Single pill regimen leads to better adherence and clinical outcome in daily practice in patients suffering from hypertension and/or dyslipidemia: results of a Meta-Analysis. High Blood Press Cardiovasc Prev 27(2):157–164. 10.1007/s40292-020-00370-5 32219670 10.1007/s40292-020-00370-5 PMC7160084

[10] Ouzzani M, Hammady H, Fedorowicz Z, Elmagarmid A (2016) Rayyan—a web and mobile app for systematic reviews. Syst Rev 5(1):210. 10.1186/s13643-016-0384-4 27919275 10.1186/s13643-016-0384-4 PMC5139140

[11] RoB 2: A revised Cochrane risk-of-bias tool for randomized trials | Cochrane Bias. Accessed August 3 (2023) https://methods.cochrane.org/bias/resources/rob-2-revised-cochrane-risk-bias-tool-randomized-trials

[12] RevMan, Web (2024) Cochrane’s systematic-review production software, is now available to the wider academic community | Cochrane. November 30, Accessed November 30, 2024. https://www.cochrane.org/news/revman-web-cochranes-systematic-review-production-software-now-available-wider-academic

[13] Cochrane Handbook for Systematic Reviews of Interventions | Cochrane Training. Accessed November 21 (2024) https://training.cochrane.org/handbook/current

[14] Altman DG, Bland JM (2005) Standard deviations and standard errors. BMJ 331(7521):903. 10.1136/bmj.331.7521.903 16223828 10.1136/bmj.331.7521.903 PMC1255808

[15] Kim BJ, Cha KS, Cho WH et al (2023) Efficacy and safety of a Single-Pill triple combination of Olmesartan, Amlodipine, and Rosuvastatin in hypertensive patients with Low-to-Moderate cardiovascular risk: A Multicenter, Randomized, Open-Label, Active-Control, phase IV clinical trial. J Cardiovasc Pharmacol Ther 28:10742484231205204. 10.1177/10742484231205204 37814541 10.1177/10742484231205204

[16] Kim TS, Rha SW, Kim SY et al (2019) Efficacy and tolerability of Telmisartan/Amlodipine and Rosuvastatin coadministration in hypertensive patients with hyperlipidemia: A phase III, Multicenter, Randomized, Double-blind study. Clin Ther 41(4):728–741. 10.1016/j.clinthera.2019.02.013 30904178 10.1016/j.clinthera.2019.02.013

[17] Jeon ES, Lim SW, Kim SY et al (2022) A randomized, double-blind, multicenter, phase III study on the efficacy and safety of a combination treatment involving Fimasartan, amlodipine, Rosuvastatin in patients with essential hypertension and dyslipidemia who fail to respond adequately to Fimasartan monotherapy. Clin Hypertens 28(1):40. 10.1186/s40885-022-00223-4 36451242 10.1186/s40885-022-00223-4 PMC9714199

[18] Jo SH, Kang SM, Yoo BS et al (2022) A prospective Randomized, Double-Blind, Multi-Center, phase III clinical trial evaluating the efficacy and safety of Olmesartan/Amlodipine plus Rosuvastatin combination treatment in patients with concomitant hypertension and dyslipidemia: A LEISURE study. JCM 11(2):350. 10.3390/jcm11020350 35054044 10.3390/jcm11020350 PMC8779537

[19] Jin X, Kim MH, Han KH et al (2020) Efficacy and safety of co-administered telmisartan/amlodipine and Rosuvastatin in subjects with hypertension and dyslipidemia. J Clin Hypertens 22(10):1835–1845. 10.1111/jch.13893 10.1111/jch.13893 PMC7692919 32937023

[20] Hong SJ, Jeong HS, Cho JM et al (2019) Efficacy and safety of triple therapy with Telmisartan, Amlodipine, and Rosuvastatin in patients with dyslipidemia and hypertension: the jeil Telmisartan, Amlodipine, and Rosuvastatin randomized clinical trial. Clin Ther 41(2):233–248e9. 10.1016/j.clinthera.2018.12.008 30665829 10.1016/j.clinthera.2018.12.008

[21] O’Meara JG, Kardia SLR, Armon JJ, Brown CA, Boerwinkle E, Turner ST (2004) Ethnic and sex differences in the Prevalence, Treatment, and control of dyslipidemia among hypertensive adults in the GENOA study. Arch Intern Med 164(12):1313. 10.1001/archinte.164.12.1313 15226165 10.1001/archinte.164.12.1313

[22] Kearney PM, Whelton M, Reynolds K, Muntner P, Whelton PK, He J (2005) Global burden of hypertension: analysis of worldwide data. Lancet 365(9455):217–223. 10.1016/S0140-6736(05)17741-1 15652604 10.1016/S0140-6736(05)17741-1

[23] Effects of ACE (2000) inhibitors, calcium antagonists, and other blood-pressure-lowering drugs: results of prospectively designed overviews of randomised trials. Lancet 356(9246):1955–1964. 10.1016/S0140-6736(00)03307-9 11130523 10.1016/s0140-6736(00)03307-9

[24] Catapano AL, Graham I, De Backer G et al (2016) 2016 ESC/EAS guidelines for the management of dyslipidaemias. Eur Heart J 37(39):2999–3058. 10.1093/eurheartj/ehw272 27567407 10.1093/eurheartj/ehw272

[25] Chobanian AV, Bakris GL, Black HR et al (2003) Seventh report of the joint National committee on Prevention, Detection, Evaluation, and treatment of high blood pressure. Hypertension 42(6):1206–1252. 10.1161/01.HYP.0000107251.49515.c2 14656957 10.1161/01.HYP.0000107251.49515.c2

[26] Hassan A, Keshk MA, Reyad M et al (2025) Bridging the gap between evidence and practice: nationwide retrospective analysis of Lipid-Modifying therapy prescription patterns in 5 million patients with type 2 diabetes mellitus. ASIDE Int Med 2(2):6–11. 10.71079/ASIDE.IM.08252522 10.71079/aside.im.08252522 PMC12425125 40949177

[27] Gami A, Blumenthal RS, McGuire DK, Sarkar S, Kohli P (2024) New perspectives in management of cardiovascular risk among people with diabetes. JAHA 13(12):e034053. 10.1161/JAHA.123.034053 38879449 10.1161/JAHA.123.034053 PMC11255726

[28] Simons LA, Levis G, Simons J (1996) Apparent discontinuation rates in patients prescribed lipid-lowering drugs. Med J Aust 164(4):208–211. 10.5694/j.1326-5377.1996.tb94138.x 8604188 10.5694/j.1326-5377.1996.tb94138.x

[29] Van Ganse E, Souchet T, Laforest L et al (2006) Long-term achievement of the therapeutic objectives of lipid-lowering agents in primary prevention patients and cardiovascular outcomes: an observational study. Atherosclerosis 185(1):58–64. 10.1016/j.atherosclerosis.2005.05.036 16038912 10.1016/j.atherosclerosis.2005.05.036

[30] Noncompliance May Cause Half of Antihypertensive Drug (1999) JAMA 282(4):313. 10.1001/jama.282.4.313 10432015 10.1001/jama.282.4.313

[31] Lee S, Yang S, Chang MJ (2021) Antihypertensive effects of Rosuvastatin in patients with hypertension and dyslipidemia: A systemic review and meta-analysis of randomized studies. PLoS ONE 16(11):e0260391. 10.1371/journal.pone.0260391 34818350 10.1371/journal.pone.0260391 PMC8612562

[32] Popat A, Yadav S (2024) Comparative effectiveness of novel combination therapies for simultaneous management of hypertension and hypercholesterolemia: A systematic review and Meta-Analysis. Cureus 16(10):e71876. 10.7759/cureus.71876 39559616 10.7759/cureus.71876 PMC11573307

[33] Strazzullo P, D’Elia L, Versiero M (2007) Response to upregulation of nitric Oxide, Inhibition of oxidative Stress, and antihypertensive effects of Statins. Hypertension 49(6). 10.1161/HYPERTENSIONAHA.107.090217 10.1161/HYPERTENSIONAHA.107.090159 17404175

[34] Briasoulis A, Agarwal V, Valachis A, Messerli FH (2013) Antihypertensive effects of statins: A Meta-Analysis of prospective controlled studies. J Clin Hypertens 15(5):310–320. 10.1111/jch.12081 10.1111/jch.12081 PMC8033902 23614844

[35] Sheraz MA, Ahsan SF, Khan MF, Ahmed S, Ahmad I (2016) Formulations of amlodipine: A review. J Pharm 2016:1–11. 10.1155/2016/8961621 10.1155/2016/8961621 PMC5086392 27822402

[36] Blank R, LaSalle J, Reeves R, Maroni J, Tarasenko L, Sun F (2005) Single-Pill therapy in the treatment of concomitant hypertension and dyslipidemia (The Amlodipine/Atorvastatin gemini Study). J Clin Hypertens 7(5):264–273. 10.1111/j.1524-6175.2005.04533.x 10.1111/j.1524-6175.2005.04533.x PMC8109673 15886529

[37] Habboush S, Sofy AA, Masoud AT et al (2022) Efficacy of Single-Pill, triple antihypertensive therapy in patients with uncontrolled hypertension: A systematic review and Meta-analysis. High Blood Press Cardiovasc Prev 29(3):245–252. 10.1007/s40292-022-00511-y 35325410 10.1007/s40292-022-00511-y

[38] Zaman MA, Awais N, Satnarine T et al (2023) Comparing Triple Combination Drug Therapy and Traditional Monotherapy for Better Survival in Patients With High-Risk Hypertension: A Systematic Review. *Cureus*. Published online July 5. 10.7759/cureus.41398 10.7759/cureus.41398 PMC10401897 37546040

[39] Lezama-Martinez D, Elena Hernandez-Campos M, Flores-Monroy J, Valencia-Hernandez I, Martinez-Aguilar L (2021) Time-Dependent effects of individual and combined treatments with Nebivolol, Lisinopril, and Valsartan on blood pressure and vascular reactivity to angiotensin II and norepinephrine. J Cardiovasc Pharmacol Ther 26(5):490–499. 10.1177/10742484211001861 33779339 10.1177/10742484211001861

[40] Alluhabi SI, Alkreathy HM, Alharthi TS et al (2023) Efficacy and safety of single pill combination of amlodipine and Valsartan in hypertensive Saudi patients. Eur Rev Med Pharmacol Sci 27(2):773–786. 10.26355/eurrev_202301_31085 36734733 10.26355/eurrev_202301_31085

