# Supplementary material for: Safety and efficacy of triple combination therapy in hypertension and dyslipidemia: a systematic review and meta-analysis of randomized controlled trials
Source: Egypt Heart J. 2026 Feb 16;78:9. doi: 10.1186/s43044-026-00720-z (PMC12909691; doi:10.1186/s43044-026-00720-z)
Supplement: Supplementary file 1 — Supplementary Material 1 [file 43044_2026_720_MOESM1_ESM.docx]

**Supplementary material:**

**Contents:**

Tables.

**1. Supplementary Table 1**: Search strategy

**2. Supplementary Table 2:** Summary characteristics of the included studies.

Figures.

Supplementary Figure. 1: Risk of bias assessment of the included trials.

**1. Triple therapy vs. ARBs plus amlodipine**

Supplementary Figure. 2: Sensitivity analysis to the change in msSBP outcome

Supplementary Figure. 3: Sensitivity analysis to the change in msDBP outcome

Supplementary Figure. 4: Sensitivity analysis to the precent change in LDL-C outcome

Supplementary Figure. 5: Forest plot of triglycerides change after 8 weeks

Supplementary Figure. 6: Forest plot of total adverse events

Supplementary Figure. 7: Forest plot of adverse drug events

Supplementary Figure. 8: Forest plot of CNS adverse events

**2. Triple therapy vs. ARBs plus Statins**

Supplementary Figure. 9: Sensitivity analysis to the change in msSBP outcome

Supplementary Figure. 10: Sensitivity analysis to the change in msDBP outcome

Supplementary Figure. 11: Sensitivity analysis to the change in HDL-C outcome

Supplementary Figure. 12: Forest plot of triglycerides change after 8 weeks

Supplementary Figure. 13: Forest plot of total adverse events

Supplementary Figure. 14: Forest plot of adverse drug events

Supplementary Figure. 15: Forest plot of CNS adverse events

**Supplementary Table 1:** Search strategy

| Database | Search strategy | N |
| --- | --- | --- |
| PubMed/Medline | ((Telmisartan OR Pritor OR BIBR OR Micardis OR Kinzalmono OR Semintra OR tolura) AND (Amlodipine OR Amlodis OR Astudal OR Norvasc OR Istin OR Amlor) AND Rosuvastatin OR Crestor OR Ezallor) AND (Dyslipidemia OR Dyslipoproteinemia OR Hyperlipidemia OR Hypercholesterolemia OR Hyperlipoproteinemia OR Hypertriglyceridemia OR atherosclerosis  AND (Hypertension OR High Blood Pressure)) | 135 |
| Cochrane | ((Telmisartan OR Pritor OR BIBR OR Micardis OR Kinzalmono OR Semintra OR tolura) AND (Amlodipine OR Amlodis OR Astudal OR Norvasc OR Istin OR Amlor) AND Rosuvastatin OR Crestor OR Ezallor) AND (Dyslipidemia OR Dyslipoproteinemia OR Hyperlipidemia OR Hypercholesterolemia OR Hyperlipoproteinemia OR Hypertriglyceridemia OR atherosclerosis  AND (Hypertension OR High Blood Pressure)) | 136 |
| Scopus | ((Telmisartan OR Pritor OR BIBR OR Micardis OR Kinzalmono OR Semintra OR tolura) AND (Amlodipine OR Amlodis OR Astudal OR Norvasc OR Istin OR Amlor) AND Rosuvastatin OR Crestor OR Ezallor) AND (Dyslipidemia OR Dyslipoproteinemia OR Hyperlipidemia OR Hypercholesterolemia OR Hyperlipoproteinemia OR Hypertriglyceridemia OR atherosclerosis  AND (Hypertension OR High Blood Pressure)) | 414 |
| WOS | ((Telmisartan OR Pritor OR BIBR OR Micardis OR Kinzalmono OR Semintra OR tolura) AND (Amlodipine OR Amlodis OR Astudal OR Norvasc OR Istin OR Amlor) AND Rosuvastatin OR Crestor OR Ezallor) AND (Dyslipidemia OR Dyslipoproteinemia OR Hyperlipidemia OR Hypercholesterolemia OR Hyperlipoproteinemia OR Hypertriglyceridemia OR atherosclerosis  AND (Hypertension OR High Blood Pressure)) | 331 |

**Supplementary Table 2**: summary characteristics of the included studies.

| Study ID | Location | Year | Study Design | Population | Intervention | Outcome | Key Findings |
| --- | --- | --- | --- | --- | --- | --- | --- |
| Kim 2023 | Korea | From August 2019 to July 2021. | RCT | Patients with hypertension and at low-to-moderate cardiovascular risk. | Olmesartan / Amlodipine / Rosuvastatin 20/5/5 mg | Percent change in low-density lipoprotein cholesterol (LDL-C) levels at from baseline to week 8. | The single-pill triple combination of olmesartan/amlodipine/rosuvastatin achieved greater LDL-C reduction than the dual combination of olmesartan/amlodipine, with comparable blood pressure control, safety, and adherence in hypertensive patients at low to moderate cardiovascular risk. |
|  |  |  |  |  |  |  |  |
|  |  |  |  |  |  |  |  |
| Jeon 2022 | NA | From July 31, 2017, to December 7, 2018. | RCT | Patients aged 19–70 years with hypertension and dyslipidemia. | Fimasartan 60 mg/ Amlodipine 10 mg + Rosuvastatin 20 mg | Change in sitting systolic blood pressure (sitSBP) and LDL-C levels from baseline to week 8. | The fimasartan/amlodipine + rosuvastatin combination effectively and safely lowers blood pressure and improves lipid levels in patients with essential hypertension and dyslipidemia inadequately controlled by fimasartan monotherapy. |
|  |  |  |  |  |  |  |  |
|  |  |  |  |  |  |  |  |
| Jo 2022 | Korea | From January 2017 to April 2018. | RCT | Patients aged 20–80 years with hypertension and dyslipidemia. | Olmesartan 40 mg/Amlodipine 10 mg (SPC) + Rosuvastatin 20 mg | Change in sitSBP from baseline and percentage change LDL-C from baseline. | The olmesartan/amlodipine/rosuvastatin triple therapy is a safe and effective option for lowering blood pressure and LDL-C, and may enhance medication adherence in patients with comorbid conditions. |
|  |  |  |  |  |  |  |  |
|  |  |  |  |  |  |  |  |
| Jin 2020 | Korea | NA | RCT | Patients aged ≥19 years, drug-free, with uncontrolled hypertension and dyslipidemia. | Telmisartan 80 mg/Amlodipine 5 mg + Rosuvastatin 20 mg | Mean sitSBP and mean LDL-C reduction in the two groups. | The telmisartan/amlodipine 80/20 mg plus rosuvastatin 20 mg combination significantly reduces blood pressure and improves lipid control in hypertensive patients with dyslipidemia, with potential to enhance long-term compliance through convenient, cost-effective therapy. |
|  |  |  |  |  |  |  |  |
|  |  |  |  |  |  |  |  |
| Hong 2019 | Korea | September 2016 through July 2017 | RCT | Korean men and women aged >19 years with primary hypertension and hypercholesterolemia requiring medical treatment | Telmisartan/Amlodipine 80 mg/10 mg +  Rosuvastatin 20 mg | Primary end point: mean change in msSBP and mean percentage change in LDL-C. Secondary end points were mean percentage change from baseline in LDL-C, total cholesterol, triglycerides, HDL-C, none HDL-C, Apo B, and Apo A1, hs-CRP, LDL-C control rate, BP target rate. | The fixed-dose combination of telmisartan, amlodipine, and rosuvastatin effectively lowered BP and LDL-C, showing safety and tolerability similar to telmisartan with amlodipine or rosuvastatin. |
|  |  |  |  |  |  |  |  |
|  |  |  |  |  |  |  |  |
| Kim 2019 | Korea | November 2015 to June 2017. | RCT | Male or female patients aged over 19 years with hypertension and dyslipidemia | Telmisartan/Amlodipine 80/10 mg + Rosuvastatin 20 mg | Primary end points were percentage changes in LDL-C level, and the mean changes in sitSBP. Secondary end points were the percentage changes in total cholesterol, TG, and HDL-C levels; mean change in sitDBP; percentage of patients achieving BP control goals and LDL-C goal. | Triple combination has value for hypertensive patients with hyperlipidemia in terms of convenience, tolerability, and efficacy. |
|  |  |  |  |  |  |  |  |
|  |  |  |  |  |  |  |  |
| Lee 2017 | Korea | September 2014 to June 2016 | RCT | Male or non–childbearing female patients aged 19–75 years with hypertension and dyslipidemia, a sitting diastolic blood pressure (SiDBP) ≥90 mm Hg, LDL-C ≤250 mg/dL, and triglycerides ≤400 mg/dL at screening. | Amlodipine 5 mg/ Losartan Potassium 100 mg/ Rosuvastatin 20 mg | Primary efficacy variables were the percent change in LDL-C level and the mean change in SiDBP. Secondary efficacy variables were the percent changes in total cholesterol, triglyceride, and HDL-C levels; the mean changes in SiSBP; the proportion of patients achieving BP goals. | Triple therapy with amlodipine, losartan, and rosuvastatin may serve as an effective treatment approach for patients with both hypertension and dyslipidemia. |
|  |  |  |  |  |  |  |  |
|  |  |  |  |  |  |  |  |
| Telmisartan, (TEL); Amlodipine, (ALD or Amlo); Rosuvastatin, (RSV or Rosu); Atorvastatin, (ATV); Olmesartan, (olme); fmasartan, (FMS); Randomised controlled trial (RCT). | | | | | | | |

**Figures**

**
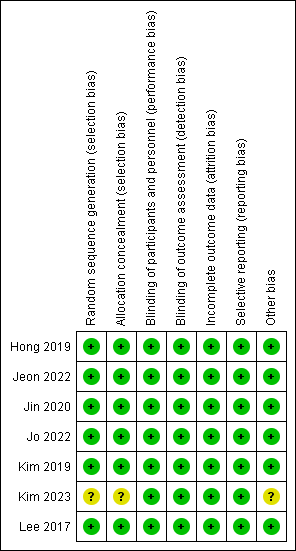
**

Supplementary Figure. 1: Risk of bias assessment of the included trials.

**
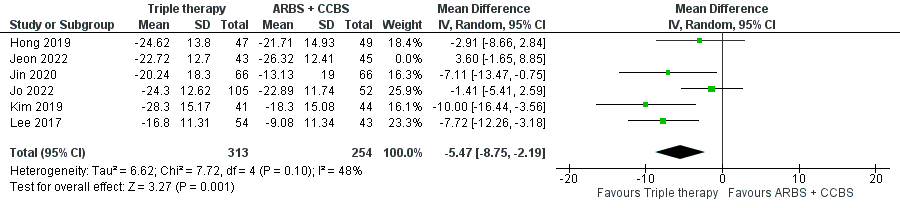
**

Supplementary Figure. 2: Sensitivity analysis to the change in msSBP outcome

**
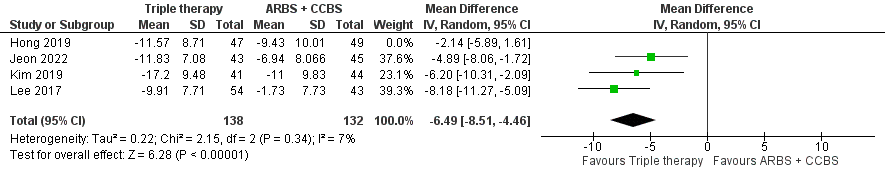
**

Supplementary Figure. 3: Sensitivity analysis to the change in msDBP outcome

**
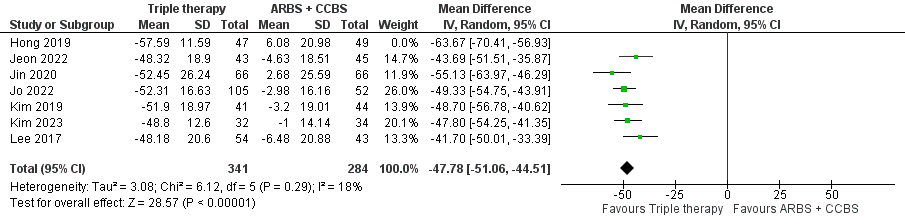
**

Supplementary Figure. 4: Sensitivity analysis to the precent change in LDL-C outcome


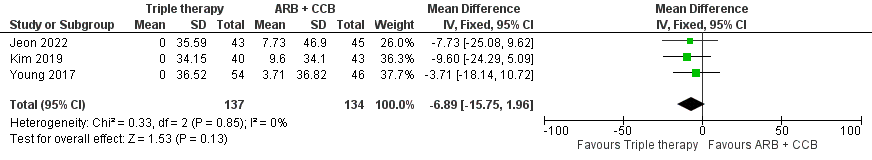


Supplementary Figure. 5: Forest plot of triglycerides change after 8 weeks

**
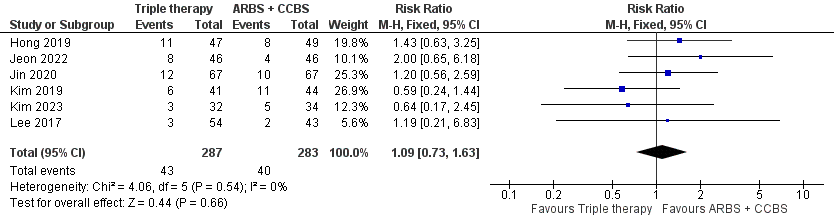
**

Supplementary Figure. 6: Forest plot of total adverse events

**
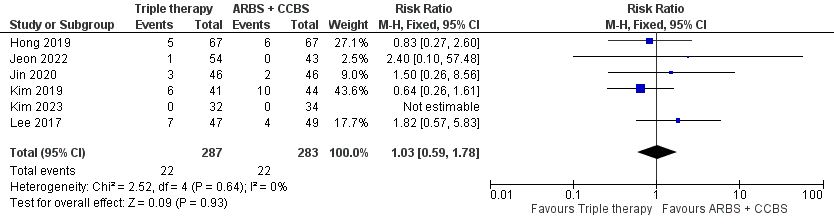
**

Supplementary Figure. 7: Forest plot of adverse drug events

**
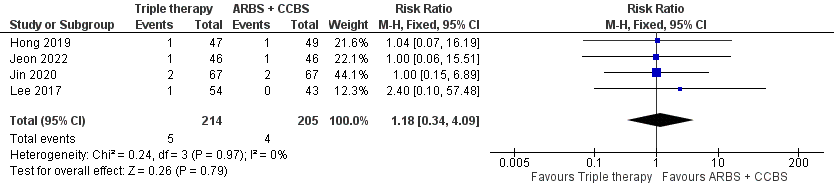
**

Supplementary Figure. 8: Forest plot of CNS adverse events

**
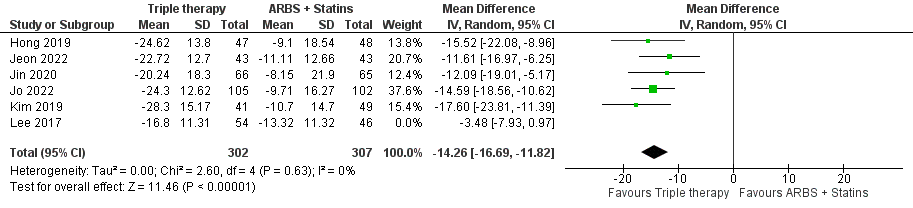
**

Supplementary Figure. 9: Sensitivity analysis to the change in msSBP outcome

**
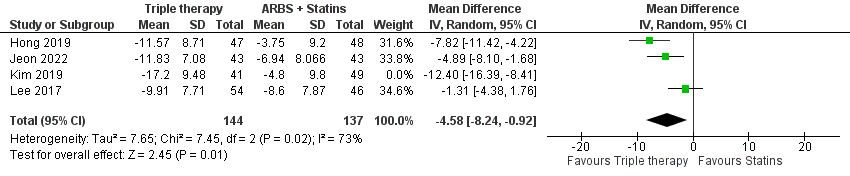
**

Supplementary Figure. 10: Sensitivity analysis to the change in msDBP outcome

**
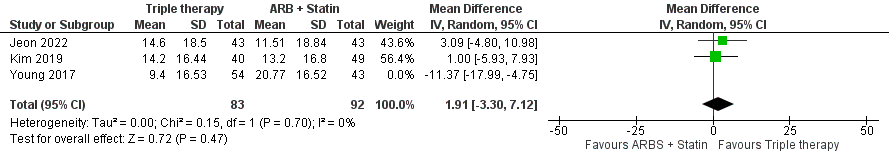
**

Supplementary Figure. 11: Sensitivity analysis to the change in HDL-C outcome

**
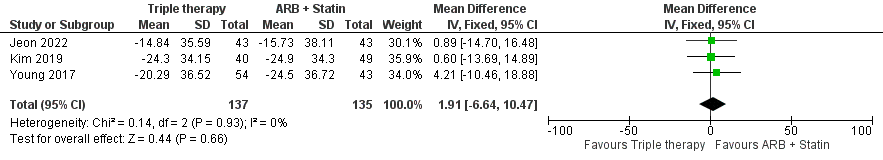
**

Supplementary Figure. 12: Forest plot of triglycerides change after 8 weeks


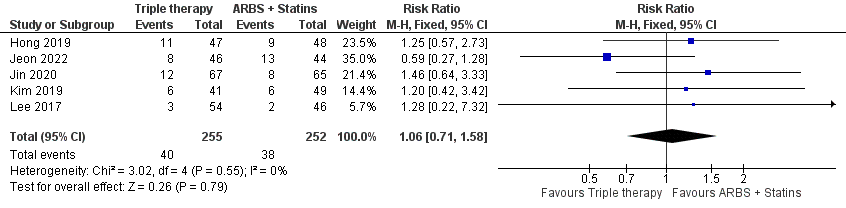


Supplementary Figure. 13: Forest plot of total adverse events

**
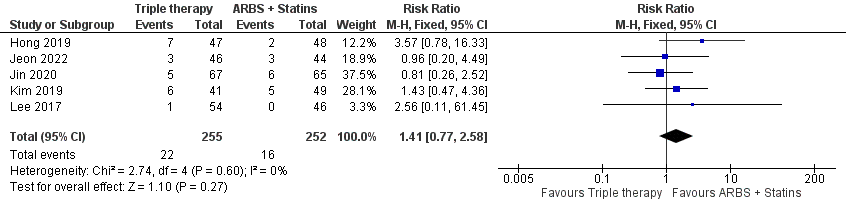
**

Supplementary Figure. 14: Forest plot of adverse drug events

**
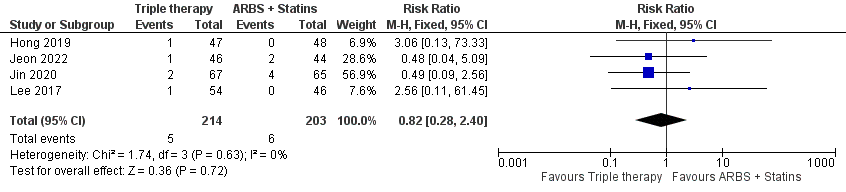
**

Supplementary Figure. 15: Forest plot of CNS adverse events
